# Practical guide for preparation, computational reconstruction and analysis of 3D human neuronal networks in control and ischaemic conditions

**DOI:** 10.1242/dev.200012

**Published:** 2022-08-05

**Authors:** Noora Räsänen, Venla Harju, Tiina Joki, Susanna Narkilahti

**Affiliations:** Faculty of Medicine and Health Technology, Tampere University, 33100, Tampere, Finland

**Keywords:** Human pluripotent stem cell, Hydrogel, Image analysis, Neuronal network, Oxygen-glucose deprivation

## Abstract

To obtain commensurate numerical data of neuronal network morphology *in vitro*, network analysis needs to follow consistent guidelines. Important factors in successful analysis are sample uniformity, suitability of the analysis method for extracting relevant data and the use of established metrics. However, for the analysis of 3D neuronal cultures, there is little coherence in the analysis methods and metrics used in different studies. Here, we present a framework for the analysis of neuronal networks in 3D. First, we selected a hydrogel that supported the growth of human pluripotent stem cell-derived cortical neurons. Second, we tested and compared two software programs for tracing multi-neuron images in three dimensions and optimized a workflow for neuronal analysis using software that was considered highly suitable for this purpose. Third, as a proof of concept, we exposed 3D neuronal networks to oxygen-glucose deprivation- and ionomycin-induced damage and showed morphological differences between the damaged networks and control samples utilizing the proposed analysis workflow. With the optimized workflow, we present a protocol for preparing, challenging, imaging and analysing 3D human neuronal cultures.

## Abstract (precis)

**Summary:** An optimized protocol is presented that allows morphological, quantifiable differences between the damaged and control human neuronal networks to be detected in three-dimensional cultures.

## INTRODUCTION

In recent years, 3D neuronal cell cultures have been developed to offer more physiologically relevant growth environments for cells than traditional 2D cultures (Cullen et al., 2011; Hopkins et al., 2015; Ylä-Outinen et al., 2019a). To utilize these 3D cultures as models for human neurological diseases and deficits or as platforms for drug development and neurotoxicity assays, the use of human-derived neuronal cells is an important factor (Baumann et al., 2016; Dragunow, 2020; Hyvärinen et al., 2019; Pires Monteiro et al., 2021; Kapr et al., 2021). To mimic the soft elastic nature of the human brain, water-retaining, polymer-based hydrogels have been successfully used as 3D scaffolds for human neuronal cells *in vitro* (Ylä-Outinen et al., 2014; Hopkins et al., 2015; Ylä-Outinen et al., 2019a). Commonly used hydrogels include the natural proteins and polysaccharides from which the tissue-specific extracellular matrix is composed. Inspired by the components and mechanical properties of brain extracellular matrix, collagens (Iwashita et al., 2019; Sood et al., 2016) and hyaluronan-based hydrogels (Karvinen et al., 2018; Samanta et al., 2022; Zhang et al., 2016) are often used in 3D neuronal *in vitro* models.

Introducing a third dimension to the *in vitro* models has challenged the currently available neuronal analysis methods, requiring them to be updated and optimized accordingly. Traditionally, image-based neuronal analysis has involved neuronal tracing, i.e. computational reconstruction of neurite centrelines (Meijering, 2010). Currently, there are multiple available tracing tools for analyzing neuronal growth in 2D (Ho et al., 2011; Ong et al., 2016) and a growing selection of tools designed for single-neuron tracing in 3D (Xiao and Peng, 2013; Zhou et al., 2018). However, tools that are suitable for tracing multi-neuron images in 3D are less abundant (Quan et al., 2016) and rarely used (Adriani et al., 2017). Consequently, there are few quantitative data describing neuronal characteristics in 3D *in vitro* models.

Commonly used metrics to describe neuronal growth in 2D cultures include neurite length, number of somas, soma size and neuronal branching complexity (Ho et al., 2011). In 3D models, these features are affected by the surrounding scaffold. Factors such as hydrogel stiffness (Cullen et al., 2011; Iwashita et al., 2019; Karvinen et al., 2018), bioactivity (Cullen et al., 2011; Evans and Gentleman, 2014) and thickness (Cullen et al., 2011) are known to influence neuronal morphology. Corresponding metrics can be used to detect growth abnormalities and the effects of damage-induced changes in neurons. For example, different forms of regulated cell death (Galluzzi et al., 2018) can cause characteristic changes in neuronal appearance. These changes include shrinking or swelling of the cell somas and degeneration and blebbing of the neurites (Gwag et al., 1999).

In this article, we present a framework for analysis of neuronal network morphology in 3D cultures. For this purpose, we cultured human induced pluripotent stem cell (hiPSC)-derived cortical neurons in 3D. First, we selected a hydrogel that optimally supported neuronal growth in long-term culture. Second, we tested software programs that were appropriate for tracing neuronal networks in 3D and selected the most suitable tool for neuronal analysis. Third, we induced neuronal network damage by exposing 3D neuronal cultures to ionomycin (IM) and oxygen-glucose deprivation (OGD). We then demonstrated quantification of differences between the damaged and intact networks by obtaining numerical data of neuronal network lengths, branching patterns and network volumes. We showed that both IM and OGD treatments reduced neuronal network properties. Our results showed that with the guidelines introduced in this study, it is possible to detect quantifiable changes in network properties with the damage inducers used and the optimized analysis workflow.

## RESULTS

### Selection of the hydrogel for neuronal culturing in 3D

**Fig. 1.**
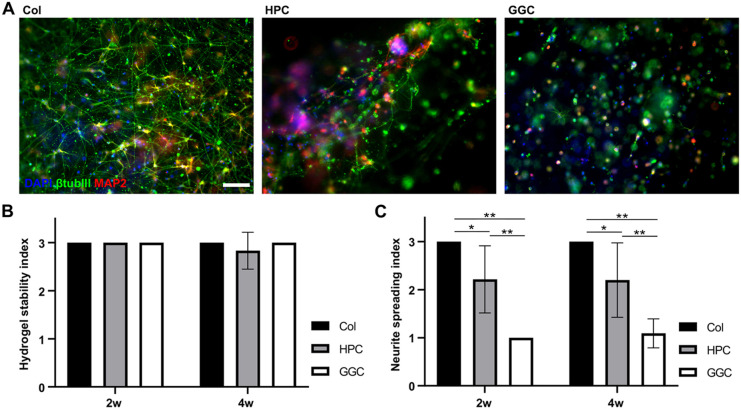
**Evaluation of neuronal network formation and hydrogel stability.** (A) Representative images of 3D neuronal networks in collagen, HPC and GGC hydrogels after 2 weeks of culturing. Scale bar: 100 µm. Images were taken with an Olympus IX51 inverted fluorescence microscope. (B) The hydrogel stability index (1, completely degraded gel; 2, partially degraded gel; 3, intact gel) was calculated at 2- and 4-week time points. The number of analyzed samples was 18 and 18 for collagen, 17 and 18 for HPC, and 18 and 18 for GGC at the 2- and 4-week time points, respectively. (C) The neurite spreading index (1, little or no neurite growth; 2, moderate neurite growth; 3, abundant neurite growth) was calculated at 2-week and 4-week time points. The number of analyzed samples was 15 and 15 for collagen, 14 and 15 for HPC, and 12 and 11 for GGC at the 2- and 4-week time points, respectively. Statistical significance was tested with the Kruskal–Wallis test followed by the Mann–Whitney *U*-test with Bonferroni correction. *P<0.05, **P<0.01. The results for B and C were collected for collagen and HPC gels from three independent experiments and for GGC gels from two independent experiments. Data are presented as mean±s.d.

To determine the optimal material for culturing hiPSC-derived cortical neurons in 3D, three different hydrogels were tested and compared for their ability to support the formation of neuronal networks during 4-week-long experiments. In addition, the stability of the hydrogels was evaluated during the culturing period. Based on our previous study (Ylä-Outinen et al., 2019a), three different hydrogels, collagen type 1 (collagen); hyaluronan-polyvinyl alcohol-collagen (HPC); and gellan gum-collagen (GGC), were selected for comparison (Fig. 1A). Both gel stability and neuronal growth were evaluated at 2- and 4-week time points.

To evaluate gel stability, we indexed gel degradability as follows: (1) completely degraded gel; (2) partially degraded gel; and (3) intact gel (Fig. S1). All collagen and GGC gel samples remained intact, whereas 17% (3/18) of the HPC gels had partially degraded by 4 weeks (Fig. 1B). The neurite spreading index (Ylä-Outinen et al., 2019a) was used to evaluate the extent of neuronal growth in each gel type (Fig. 1C). Briefly, the growth rate was indexed as follows: (1) little or no neurite growth; (2) moderate neurite growth; and (3) abundant neurite growth. We found that collagen supported neurite spreading at both time points better than HPC and GGC gels (Fig. 1C). All (18/18 at each time point) of the samples evaluated at the 2- and 4-week time points displayed abundant neurite growth. The index for collagen was significantly higher than that of the HPC and GGC gels at the 2-week time point (*P*<0.05 and *P*<0.01, respectively) and at the 4-week time point (*P*<0.05 and *P*<0.01, respectively).

In addition to the stability of the collagen gel and the superior support for neuronal growth, the preparation of this gel was more practical than the preparation of HPC and GGC gels. Given that the HPC and GGC gels were composed of two or more components that gelled quickly after mixing, non-homogenous blending of the components could not be completely avoided. Thus, the most homogenous networks were obtained with the collagen gel, which consisted of only one main component and had a longer gelling time.

In summary, in long-term cultures, collagen was the best-performing gel in terms of support for neuronal growth as it showed excellent stability and had the most uniform results of all the evaluated gels with the lowest variation between samples. Collagen was also the most practical gel to prepare, and it was therefore selected for more detailed studies.

### Staining protocol optimization

Here, our previously used immunocytochemical (ICC) staining protocol for neurons cultured in hydrogels (Koivisto et al., 2017) was further optimized to improve sample cleanness and image quality for analysis. To remove excess antibodies from the gels, washing times were prolonged from 1 to 2 days for primary antibodies and from two quick washes to 2 days of washing for secondary antibodies. With prolonged washing times, sample cleanness improved considerably (Fig. S2A,B). The gels were prepared with a thickness of ∼500 µm, as this thickness has been used for 3D neuronal culturing in several studies (Aregueta-Robles et al., 2019; Cullen et al., 2011; Madl et al., 2017) and is considered to provide a valid 3D environment for neurons (Cullen et al., 2011). Samples of this thickness could also be imaged with the confocal microscope that was used (Fig. S2C). For image processing, samples were cropped to a size of 255 µm×255 µm×100 µm. To provide sufficient details about neuronal morphology for computational reconstruction, we decided to use both βIII-tubulin and MAP2 antibodies to stain the neurites (Fig. S2).

### Software selection and comparison

Several commercial and open-source software programs for neuronal tracing were surveyed, and their suitability for the analysis of 3D neuronal networks was pre-evaluated (Table 1). The primary requirement for the software was an automatic tracing option for multi-neuron images. Considering the facility of the entire analysis workflow, image pre-processing options, trace editing tools and real-time, network-level measurements were also considered desirable features. Most of these features were found in two commercial software programs: Imaris and Avizo, which were selected for comparison. First, analysis workflows were optimized for both software programs with sample images of neuronal networks grown in collagen. For tracing, the automatic mode of the ‘Autopath’ tracing tool in Imaris and the ‘Auto skeleton’ tool in Avizo were used. The images were processed with the most suitable tools found in each software. Tracing errors and time-consuming processing steps in the workflows were identified. Additionally, the suitability of the software for measuring neuronal network lengths, branching patterns and soma-related metrics was evaluated.

**Table 1.**
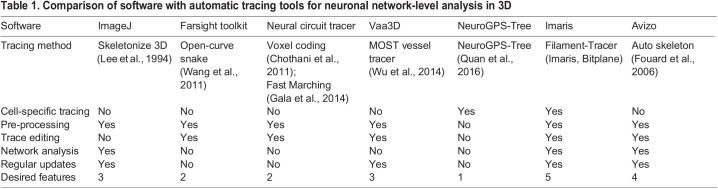
Comparison of software with automatic tracing tools for neuronal network-level analysis in 3D

**Fig. 2.**
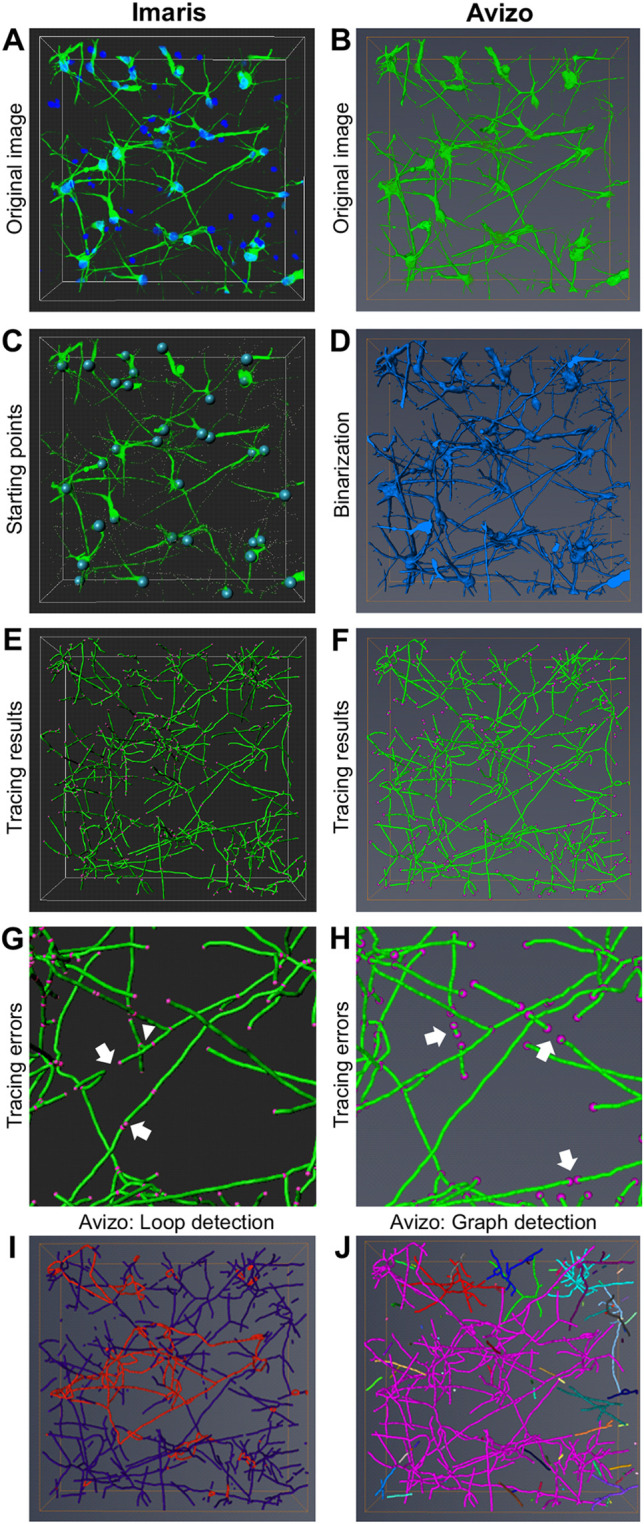
**Neuronal tracing with Imaris and Avizo.** (A,B) Original images of ICC-stained neurons before tracing with Imaris (A) and Avizo (B). (C) Neuronal somas detected as starting points with Imaris are shown as blue spheres. (D) Binarized image made with Avizo is shown in blue. (E,F) Tracing results with Imaris (E) and Avizo (F). Neurite centrelines are shown in green, and neurite branching and ending point locations are shown in magenta. (G) Tracing errors with Imaris. Neurite segments that formed bridges between neurons were randomly cut (arrows). Additionally, some uneven neurite surface structures were detected as small branches (arrowhead). (H) Tracing errors with Avizo. Arrows indicate discontinuous neurite fragments. (I) Loops created by closed paths formed by neurons in Avizo (red). (J) Single graph showing interconnected neurons detected inaccurately as a single neuron by Avizo (magenta).

The tracing tools in Imaris (Fig. 2A,C,E,G) and Avizo (Fig. 2B,D,F,H-J) employed different tracing principles. In Imaris, tracing involved the detection of somas as starting points from which tracing proceeded along the neurites (Fig. 2C). In addition, neurites with gaps or thin sections appearing as gaps were traced as continuous branches, whereas discrete neurite fragments were automatically excluded from the trace. Therefore, only neurites that contained their soma in the imaged region could be analysed. The most important parameters that were adjusted for tracing with Imaris were neurite seed point diameter and maximum gap width between neurite segments. In our dataset, the use of 1 μm diameter seed points (2× *z*-interval) enabled detection of neurites within the limits of the image resolution. The maximum gap width was set to 4 μm to enhance neurite continuity while avoiding formation of connections between closely located neurites. In Avizo, the neuronal network was traced without soma detection and gap bridging. Thus, in contrast to traces made with Imaris, disconnected neurites and neurite fragments were included in traces made with Avizo (Fig. 2F,H). However, to avoid gap formation in thin neurite sections, we pre-processed the images using a curvature-driven diffusion filter before tracing with Avizo. The filter enabled enhancement of continuous structures, such as neurites, while eliminating discontinuous background signal.

Neither βIII-tubulin nor MAP2 staining covered the neuronal somas entirely in the stained samples. This caused some of the somas to appear hollow. DAPI could not be used to fill these regions owing to the few non-neuronal nuclei in the background (Fig. 2A). Because Imaris software detected the somas based on their intensity as starting points for tracing, without high-intensity staining, the somas were sometimes not identified correctly, and the starting point positions had to be corrected manually. In Avizo, hollow somas were traced as loops that created extra length in the networks (Fig. 2I). To fill the hollow regions with Avizo, the images were binarized, and the enclosed cavities were filled using the ‘Fill holes’ module (Fig. 2D). If the cavities were not fully enclosed during binarization, the resulting loops could be detected and removed manually after tracing was complete using the ‘Filament Editor’ module.

**Fig. 3.**
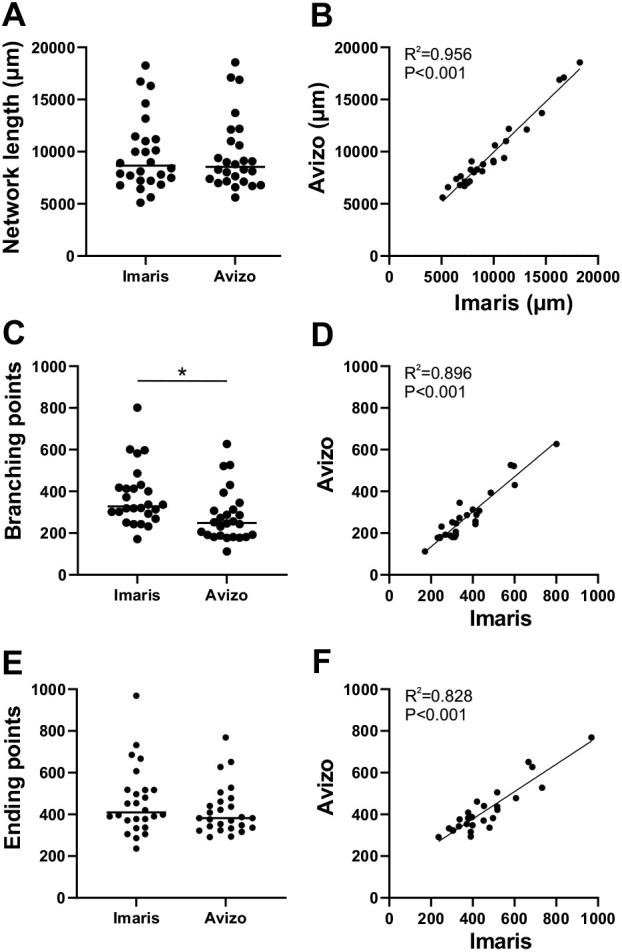
**Analysis software comparison.** (A,B) The total network lengths of trace pairs acquired with Imaris and Avizo. (C,D) Branching points acquired with Imaris and Avizo. (E,F) Ending points acquired with Imaris and Avizo. Statistical differences for A, C and E were calculated by the Mann–Whitney test (*P<0.05), and correlations for B, D and F were calculated by Spearman's rank correlation. The data were acquired from 26 samples. Horizontal lines in A, C and E indicate the mean and circles the individual data points.

To evaluate the similarity of the tracing results acquired with Imaris and Avizo, 26 images were traced with both software programs (representative traces from four images; Fig. S3). A pairwise comparison was performed for the corresponding traces in terms of total network length (Fig. 3A,B), the number of detected branching points (Fig. 3C,D), and the number of detected ending points (Fig. 3E,F). All traced neuronal processes were included in the measurements. Despite the differences in the tracing methods, a high positive correlation (*r*=0.960) was observed in the network lengths detected by the two software programs. Moreover, the network lengths were highly similar between the corresponding traces acquired with the different methods (Fig. 3B,D,F). Given that tracing errors did not significantly affect the detected network length, we concluded that both Imaris and Avizo could be used to quantify the total lengths of neuronal networks in 3D cultures.

The number of neurite branching points is a more controversial measure than neurite length because of the frequent tracing errors typically occurring in neurite crossover points (Quan et al., 2016; Ong et al., 2016). Thus, neuronal tracing tools often interpret neurite crossing points as branching points. As expected, excess branching points were created by both software programs at neurite crossover points; these excess branching points resulted in neurite cutting by Imaris (Fig. 2G) and most neurons being merged into a single neuronal tree by Avizo (Fig. 2J). Although a high positive correlation (*r*=0.913) was observed between the number of branching points detected by the two software programs (Fig. 3D), Avizo detected significantly fewer branching points than Imaris (Fig. 3C). This was likely caused by the gap-bridging feature in Imaris, which allows neurite fragments to be connected to the neuronal tree, in some cases leading to creation of excess branches. Although both Imaris and Avizo enabled manual trace editing, this was extremely time consuming and therefore impractical. Given that we observed tracing errors that specifically affected the number of neurite branching points, we decided not to use the measure for quantification of neuronal branching in the 3D cultures.

A high positive correlation (*r*=0.821) was also observed between the number of ending points detected by Imaris and Avizo (Fig. 3F). However, the correlation was slightly weaker than the correlation between the detected branching points. Unlike in case of branching points, there was no significant difference in the number of detected ending points between the software programs (Fig. 3E). Nevertheless, we did notice some relevant differences in the ending point detection between Imaris and Avizo. Because Imaris prevented neurites from connecting to more than one neuronal tree, bridges between separate neurons were cut, resulting in excess ending points (Fig. 2G). This phenomenon was most evident in the densest networks. In the case of traces acquired with Avizo, the number of neurite ending points was affected by the inclusion of disconnected neurite fragments in the trace (Figs 2H, 3E). Hence, in the shortest networks, Avizo detected more ending points than Imaris. Given that the number of detected ending points was not disproportionately affected by increasing network length in Avizo, we concluded that the measure could be used to evaluate neuronal branching in Avizo. However, the measure could not be used to separate increased neurite fragmentation from increased branching.

Although neither of the software programs included soma segmentation in the tracing workflow, Imaris was able to detect neuronal somas as spherical objects. Hence, the tracing result could be used to quantify the number of neuronal somas and to evaluate the number of neuronal processes originating from each soma. Thus, we could evaluate neuronal branching based on the number of neurite attachment points. This measure was considered more suitable for evaluating neuronal branching in 3D images than the number of ending points given that 3D images are typically limited in size and most neurites traverse outside the imaged region. Furthermore, because Imaris has been used in a few other studies for neuronal analysis *in vitro* (Adriani et al., 2017; Aregueta-Robles et al., 2019), we selected Imaris for further optimization of the analysis workflow.

### Optimization of neuronal analysis with Imaris

The most laborious step in neuronal analysis with Imaris was manual detection of the neuronal somas as starting points for tracing. Therefore, postmitotic neuronal somas were stained with NeuN (RBFOX3 – HGNC). With the staining, Imaris was able to correctly detect 85% of the starting points. As nuclear DAPI staining was localized more evenly than NeuN staining in somas, the DAPI channel was masked with NeuN staining to facilitate the selection of postmitotic neuronal nuclei. In this way, the automatic starting point detection accuracy was improved to 89%. The remaining errors were almost exclusively caused by merged somas. The image size for analysis was selected to be 255 µm×255 µm×100 µm to keep the tracing time moderate. For a dense network, this would result in a tracing time of approximately 12 min per image (CPU: Intel Core i7-6900 K, 3.2 GHz).

### Detection of differences in neuronal networks with Imaris

**Fig. 4.**
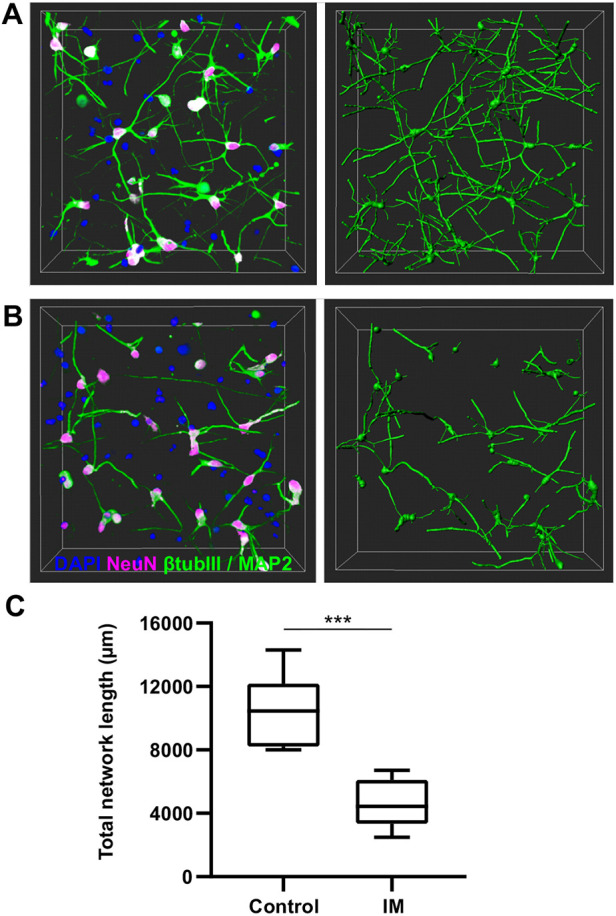
**Detection of differences in neuronal network length after IM treatment with Imaris.** (A) A representative image of the neuronal network in a control sample (left) and the tracing result (right). (B) A representative image of the neuronal network in IM-treated samples (left) and the tracing result (right) showing network length reduction. In A and B, the neuronal networks were stained for βIII-tubulin, MAP2, NeuN and DAPI. The images are 255 µm×255 µm×100 µm in size, taken with a Zeiss LSM 780 LSCM confocal microscope system. (C) Quantification of total network lengths. The number of analysed samples was eight (from four hydrogels, two images taken from each hydrogel) for both groups. Data are presented as Tukey box plots, showing whiskers for the minimum and the maximum values, box borders for the 25th and the 75th percentile, and the middle line for the median value. Statistical differences were calculated using the unpaired two-tailed Student's *t*-test (***P<0.001).

To demonstrate detection of differences in neuronal network characteristics, 3D neuronal cultures were treated with IM to create a set of samples representing neuronal damage. Subsequently, Imaris was used for tracing and analysis of the networks in control (Fig. 4A) and IM-treated (Fig. 4B) samples. Cells that expressed small amounts of NeuN but did not have growing neurites were excluded from the analysis. As a result, a significant reduction in the total network lengths between the samples treated with IM and control samples (*P*<0.001; Fig. 4C) was detected. The total network length was on average 10,500 µm in the control samples and 4600 µm in the IM-treated samples; the difference in these values corresponded to a 56% network length reduction after IM treatment.

### Neuronal exposure to OGD

Next, we studied whether Imaris was able to detect differences in neuronal networks exposed to OGD. To model the OGD-induced damage, neurons were exposed to low oxygen (1% O_2_) and cultured in glucose-free medium for 24 h as previously done in 2D culture conditions (Juntunen et al., 2020). After OGD, neurons were subjected to 24 h reperfusion in normoxic conditions. In addition, both untreated and IM-treated control samples were prepared. After exposure and ICC staining, the neuronal networks were traced and analysed with Imaris, and the results were compared in terms of selected metrics. The timeline for the sample preparation and exposure is summarized in Fig. 7.

**Fig. 5.**
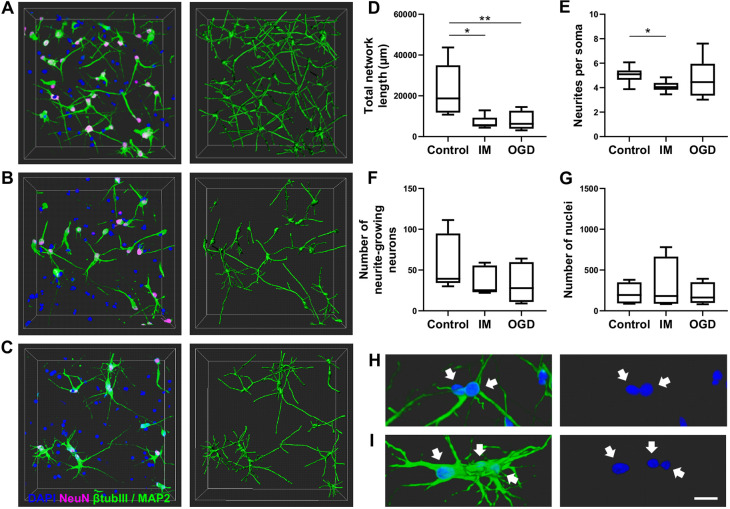
**Analysis of neuronal networks exposed to IM and OGD.** (A-C) Representative images of the neuronal network (left) and the tracing result (right) for a control sample (A), a sample treated with IM (B) and a sample exposed to OGD (C). Neuronal networks were stained for βIII-tubulin, MAP2, NeuN and DAPI. The images are 255 µm×255 µm×100 µm in size and were imaged with a Zeiss LSM 780 LSCM confocal microscope system. (D-G) Quantification of total neuronal network lengths (D), the average number of neurites per soma (E), the number of neurite-growing neurons (F) and the number of nuclei (G). The number of analyzed samples was ten (from two experiments, each sample including two or three technical replicates) for all groups. Data are presented as Tukey box plots, showing whiskers for the minimum and the maximum values, box borders for the 25th and the 75th percentile, and the middle line for the median value, and analyzed statistically using the Kruskal–Wallis with Dunn's multiple comparisons test. *P<0.05, **P<0.01. (H,I) Merged somas in a control sample (H) and soma swelling in an OGD sample (I). White arrows point to nuclei. Scale bar: 20 µm.

First, we were able to show a reduction in total network length in the IM-treated (Fig. 5B) and OGD-treated (Fig. 5C) samples in comparison to the total network length of the control samples (Fig. 5A). On average, the total network length decreased significantly from 23,000 µm in control samples to 7200 µm (69% reduction; *P*<0.05) in IM-treated samples and to 7800 µm (66% reduction; *P*<0.01) in OGD-treated samples (Fig. 5D). Second, the neuronal branching patterns were compared among the three groups by quantifying the number of neurites originating from neuronal somas. Here, we were able to show a significant difference in the average number of neurites per soma in IM-treated samples in comparison with the control samples (Fig. 5E). On average, the number of neurites per soma was 5.0 in the control samples, 4.1 (19% reduction, *P*<0.05) in the IM-treated samples and 4.7 (6% reduction) in the OGD-exposed samples.

Third, the number of viable, neurite-growing neurons was compared between each group. As a result, there was a reduction in the number of neurite-growing neurons in both groups of exposed samples in comparison with control samples (Fig. 5F). However, this reduction was not significant because the variation between the samples was high. The number of neurite-growing neurons was on average 60 in the control samples, 35 (41% reduction) in the IM-treated samples and 33 (46% reduction) in the OGD-treated samples. Despite the changes in the number of neurite-growing neurons, the overall cell number did not differ significantly among the groups (Fig. 5G). Interestingly, soma swelling was observed in the OGD-treated samples. This swelling caused closely located somas to merge into larger cell clusters (Fig. 5H,I). As soma clustering complicates starting point detection in Imaris, inspection and correction of the soma positions was performed partly manually for the analysis.

### Analysis of network volumes

**Fig. 6.**
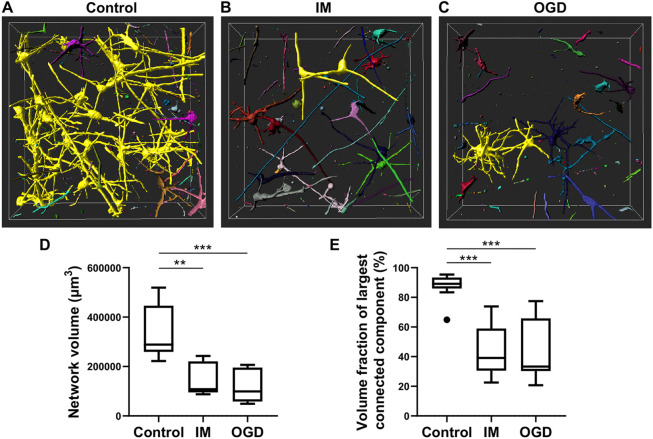
**Analysis of neuronal volume and connectedness.** (A-C) Volume reconstructions of a neuronal network in a control sample (A), a sample treated with IM (B) and a sample exposed to OGD (C). The largest connected component is labelled in yellow. (D) Quantification of total network volume (µm^3^) in the neuronal networks. (E) Quantification of network connectedness as the volume of the largest connected component. The data in D and E were acquired from ten samples (from two experiments, each sample including two or three technical replicates) for all groups. Data are presented as Tukey box plots, showing whiskers for the minimum and the maximum values, box borders for the 25th and the 75th percentile, and the middle line for the median value. Statistical tests were performed with the Kruskal–Wallis with Dunn's multiple comparisons test; **P<0.01, ***P<0.001. Black circle indicates an outlier.

In addition to the commonly used measures, including neuronal network lengths and branching patterns (Ho et al., 2011), measures that could be used to further characterize dense neuronal networks were considered. Imaris could not distinguish neurite branching points from crossing points, preventing detailed morphological analysis of individual neurons. We also wanted to focus the analysis on network-level phenomena. Neurons *in vitro* have a fundamental tendency to connect with other neurons and thereby form similar interconnected networks than *in vivo* (Kirwan et al., 2015). Furthermore, locations in a network where axons and dendrites cross closely offer potential places for synapses (Stepanyants and Chklovskii, 2005). Therefore, a measure for network connectedness was defined as the fraction of volume or length of the network that forms the largest connected component. With Imaris, surface reconstructions of the networks in IM-treated, OGD-treated and control samples were created to measure the total network volumes and volume fractions of the reconstructed objects (Fig. 6A-C). Here, the total network volumes followed a similar trend to previously measured network lengths, as IM-treated (*P*<0.01) and OGD-treated (*P*<0.001) networks had significantly lower volumes than the control networks (Fig. 6D). Finally, significantly greater connectedness was detected in the control networks in comparison with IM-treated and OGD-treated networks (Fig. 6E; *P*<0.001 for both) was shown. Here, the connectedness of the networks in control samples was on average 88% and was reduced to 43% in the networks in IM-treated samples and to 44% in the networks in OGD-treated samples.

### Analysis workflow summary

**Fig. 7.**
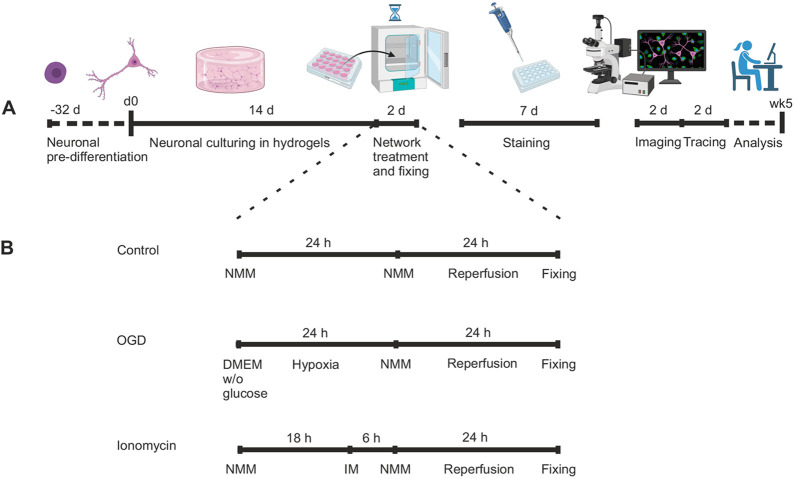
**Timeline for experimental design.** (A) Representative timeline for the preparation, treatment and analysis of a 3D neuronal *in vitro* model. One round of the experimental setup took ∼5 weeks. Prior to that, neurons were pre-differentiated for 32 days in 2D and then cultured for 2 weeks inside hydrogels. OGD and control treatments were performed within 2 days, and the staining protocol was performed within 7 days. Imaging and neuronal tracing were performed in ∼4 days. d, days; wk, week. (B) Timeline for the control, OGD and IM treatments. The samples were exposed to OGD for 24 h and IM treatment for 6 h. Neural maturation media was changed for all the samples 24 h before fixing. Illustrations were created with BioRender.com.

Altogether, one experimental round of neuronal culturing, exposure, staining, imaging and analysis was conducted over 5 weeks starting from cell encapsulation in hydrogels (Fig. 7A). After 2 weeks of culture in collagen gels, we exposed the neurons to either 24 h of OGD or 6 h of IM treatment following 24 h of re-perfusion (Fig. 7B). After prolonging the antibody washing times, ICC staining of neurons in hydrogels was performed, which took 7 days. Imaging and analysis of 30 images took 4 days.

## DISCUSSION

Here, we show that neuronal networks can be traced and analysed in 3D with commercially available analysis tools during normal and challenged culture conditions. First, we evaluated the properties of hydrogels in relation to their stability and ability to support the formation of neuronal networks during a 4-week experimental setting. Also, we showed that collagen I is an excellent scaffold material for 3D human neuronal cultures. Here, collagen performed better than HPC and GGC gels in terms of neuronal growth and stability. There is also previous evidence of collagen suitability for neuronal tissue engineering (Sood et al., 2016; Iwashita et al., 2019; Lam et al., 2020; Ylä-Outinen et al., 2019a). Collagen is also abundantly present in the extracellular matrix of the developing foetal brain (Medberry et al., 2013; Sood et al., 2016, 2019). Stability is an important factor for the gels to withstand the long cell-culturing periods as well as multiple washes and long incubation times that are required during the staining process. Especially at low concentrations, hydrogels may degrade as a result of extensive washing and incubation (Aregueta-Robles et al., 2019). Previously, we tested collagen type 1 products from Cultrex (Trevigen; Ylä-Outinen et al., 2019a) and Corning (L. Ylä-Outinen and S.N.; unpublished data) as 3D neuronal culturing scaffolds. These products did not give as stable a result during a prolonged culturing time of 4 weeks as the product that was used in this study (Gibco). Thus, there might be differences in product specifications between different suppliers, as well as batch-to-batch variation. However, in this work, all the studied gels showed good stability during the experiments.

Neurons of human origin were used in this study. Considering disease modelling and drug development, human cells are particularly important, as there are differences in the development and function of human- and animal-derived neuronal cells (Baumann et al., 2016; Dragunow, 2020; Hyvärinen et al., 2019; Ylä-Outinen et al., 2019b). However, most of the previous knowledge of neuronal network damage after stressful treatments is based on studies using 2D cultures of animal-derived neurons (Gwag et al., 1999; Abramov et al., 2007; Chen et al., 2019). Brain organoids are one option for culturing human neuronal cells in 3D (Kim et al., 2021). However, modelling and analysing neuronal networks in 3D is more convenient and less laborious to perform with 3D hydrogel models. Currently, there are still few reports describing the culturing of hiPSC-derived neurons in 3D scaffolds (Nikolakopoulou et al., 2020; Ylä-Outinen et al., 2019a). Here, we were able to show maturing and stable culturing of hiPSC-derived neurons in 3D scaffolds for 4 weeks. This time frame is considered long enough to obtain mature neuronal networks (Hyvärinen et al., 2019) and short enough from the model usability perspective for future studies.

We surveyed several commercial and open-source software programs for neuronal tracing and evaluated their suitability for the analysis of 3D neuronal networks. The primary requirement for the software was an automatic tracing option for multi-neuron images. Additionally, features such as image pre-processing options, trace-editing tools and real-time network-level measurements were considered desirable. Most of the surveyed tools were designed for tracing single neurons. Many of these tools (Xiao and Peng, 2013; Zhou et al., 2018) are available in the open-source software Vaa3D (Long et al., 2010). For analysing neuronal networks, we previously found suitable pre-processing (Narayanaswamy et al., 2011), tracing (Wang et al., 2011) and trace-editing (Luisi et al., 2011) tools from the open-source software Farsight toolkit, which was no longer available at the time of this study. We also found a promising tracing tool in the open-source software NeuroGPS-Tree (Quan et al., 2016), which enabled neuronal tracing, leaving analysis and processing to be performed separately. However, all the desired features were found in two commercial software programs: Imaris (Bitplane) and Avizo (Thermo Fisher Scientific/ZIB), which were selected for further testing.

Both Imaris and Avizo could be used to trace 3D neuronal networks and to obtain versatile data describing network morphology. Despite the differences in the tracing methods employed by these two software programs, the quantified network properties were not drastically different between the traces obtained with the software. However, owing to the small size of the analysed regions in our samples, most neurons had neurites that traversed outside the images, preventing the full neuronal branching complexity from being captured. We also detected tracing errors in neurite branching points causing inaccuracy in most branching-related measures with both software programs. Errors in neurite crossing and branching points are a common issue in multi-neuron traces (Ong et al., 2016), as we showed with both Imaris and Avizo. Some tracing methods are designed to prevent errors by pruning excess connections, predicting neurite direction (Quan et al., 2016), preventing loop formation, or otherwise considering optimal neuronal wiring patterns (Cuntz et al., 2010). A feature preventing loop formation was also incorporated in the tracing tool in Imaris but was not useful in preventing errors in dense cultures. Instead, the feature caused some of the tracing errors by wrongly assuming the neuronal path. Although the number of neurite ending points is a measure that can be associated with neuronal branching patterns (Ho et al., 2011), we decided not to use this measure to describe neuronal branching in dense 3D networks because of restrictions in image volumes and differences in tracing methods.

As Imaris has a feature for soma detection and is more frequently used for studying neuronal network properties *in vitro* than Avizo, we chose to use Imaris to optimize the analysis workflow. The detection of somas in Imaris allowed us to use the number of neurite attachment points to a soma as a reliable measure of neuronal branching. Although we selected Imaris for neuronal analysis in this study, different analysis tools are suitable for different applications. For instance, Avizo would be a preferable choice for quantification of neuronal network lengths from images that do not contain somas, as in microdevices with restrictive chambers. In addition to the tested commercial software, the open-source software ImageJ contains tracing (Lee et al., 1994) and analysis (Arganda-Carreras et al., 2010) tools that could be used for analysing interconnected networks. In addition, ImageJ can be used to analyse neuronal directionality and orientation in a robust manner and is free to use (Honkamäki et al., 2021).

To quantify morphological changes in neuronal networks with the selected analysis method, we exposed our neuronal model to stressful treatments. Although cell death is typically used as an indicator of damage (Abramov et al., 2007; Nierode et al., 2016), morphological alterations in neuronal networks can be used to describe changes in network properties in response to insults and subsequently in response to therapeutic compounds. With exposure to IM, we were able to validate the analysis method and quantify reduced network lengths in IM-treated networks in comparison with control samples. The Imaris-based analysis method was further used to quantify the differences of OGD- and IM-induced damage in neuronal networks. The OGD treatment used in this study has been used to model ischaemic stroke *in vitro* (Abramov et al., 2007; Juntunen et al., 2020; Ristola et al., 2021) and *in vivo* (Cui et al., 2017). With the selected metrics describing network length, branching, volume and connectedness, we were able to show robust morphological differences between the networks in IM- and OGD-treated samples and those in control samples. As a result, we showed significantly reduced neuronal network properties in both IM- and OGD-treated cultures in comparison with control cultures. The results of this study are encouraging and provide motivation to continue such studies for other biological questions.

Despite the successful comparison of the network properties with Imaris, the detection of neuronal somas could not be performed fully automatically because of cell clustering and soma swelling, especially in the OGD-treated samples. In the future, starting-point detection could be facilitated by increasing the contrast between the nuclear channel and the neurites or by including a feature in Imaris that would enable starting-point detection from the nuclear channel separately. Green fluorescent protein (GFP) is often used to visualize neurons for tracing and more efficiently labels neuronal somas for starting-point detection than the ICC markers used in this study (Quan et al., 2016). However, neural progenitor cells and astrocytes typically co-develop with neurons in human pluripotent stem cell-derived neuronal cultures (Hyvärinen et al., 2019) during the prolonged period of human neurogenesis. Thus, GFP expression in non-neuronal cells complicates the specific detection of neurons. Although some of the non-neurite-growing neurons in the samples could be proliferating neural progenitor cells or dead cells trapped inside the hydrogels, we found no difference in the total number of nuclei among the conditions. In addition to the quantified measures, OGD-treated neurons exhibited soma swelling, a phenomenon that was not characteristic of the IM-treated samples.

Morphological characterization of neuronal networks is necessary to observe the effects of therapeutic interventions when utilizing these models for drug screening and development and for cell-based therapy development. Although neurite length and branching complexity are common metrics to describe neuronal network morphology, network skeletonization is required to obtain these measures. We noted that when tracing is inconvenient, network volume-based measures can be used as simple but robust measures of network differences. Whether utilizing tracing or volume-based analysis techniques, performing the analysis of 3D cultures in 3D is important to detect changes in neuronal network morphology reliably. Although 2D projection images have been used for high-throughput analysis of 3D neuronal networks (Spijkers et al., 2021; Wevers et al., 2016), this approach would complicate the analysis of neuronal branching, as the somas of neurons that grow on top of each other overlap in the projections. Similarly, the connectedness measure that was used to evaluate neuronal network integrity in 3D could not be used for 2D projections.

Here, with the optimized analysis workflow in Imaris, we were able to show quantifiable differences in network properties in 3D neuronal cultures subjected to different challenges. Notably, we showed morphological alterations in neuronal networks after an OGD insult for the first time in a 3D hiPSC-derived neuronal model. This workflow could be further used in disease modelling and drug screening in 3D neuronal models.

## MATERIALS AND METHODS

### hiPSCs and neuronal differentiation

The hiPSC line 10212.EURCCs (Kiamehr et al., 2019) was generated with Sendai virus technology (Life Technologies) (Ojala et al., 2015) at the Faculty of Medicine and Health Technology (MET), Tampere University, Finland. The hiPSCs used in this study were acquired from voluntary subjects who had provided written and informed consent. The institute has a supportive statement from Pirkanmaa Hospital District for the generation of hiPSCs from donor cells (R12123) and to use generated cell lines in neuronal research (R05116). Gene and protein expression as well as the karyotype of the hiPSC line were under frequent quality control assessment. Mycoplasma assays were also performed regularly.

hiPSCs were expanded in a feeder-free culture system as described previously (Hongisto et al., 2017), and cortical neurons were differentiated as previously described (Hyvärinen et al., 2019). Neuronal differentiation before hydrogel encapsulation was performed in laminin-coated wells (15 μg/μl, laminin LN521, BioLamina). Briefly, the neural maintenance medium (N3) consisted of 1:1 DMEM/F12 with GlutaMAX and Neurobasal medium and was supplemented with 0.5% N2, 1% B-27 with retinoic acid (RA), 0.5 mM GlutaMAX, 0.5% non-essential amino acid solution, 50 μM 2-mercaptoethanol (all purchased from Thermo Fisher Scientific), 2.5 μg/ml insulin (Merck KGaA), and 0.1% penicillin-streptomycin (Thermo Fisher Scientific). For neural induction, the medium was supplemented with 100 nM LDN193189 (Merck KGaA) and 10 μM SB-431542 (Merck KGaA) for 12 days. For the neural proliferation stage, the medium was supplemented with 20 ng/ml fibroblast growth factor-2 (FGF2, R&D Systems) for 13 days. Thereafter, the cells were cultured in neural maturation medium (NMM) consisting of N3 medium supplemented with 20 ng/ml brain-derived neurotrophic factor (BDNF, R&D Systems), 10 ng/ml glial-derived neurotrophic factor (GDNF, R&D Systems), 500 μM dibuturyl-cyclic AMP (db-cAMP, Merck KGaA) and 200 μM ascorbic acid (Merck KGaA) for 7 days to promote neuron maturation.

On day 32, the cells were encapsulated in hydrogels. Cells were detached from well plates using Accutase (Life Technologies), centrifuged (3 min, 300 ***g***) and resuspended twice, and encapsulated inside hydrogels at a final concentration of 5×10^6^ cells/ml with NMM for further maturation. During the first day of cell culture in the hydrogels, the medium was supplemented with 2 µl/ml Rho kinase inhibitor (ROCKi, Merck KGaA). Cells were cultured in the gels for either 2 or 4 weeks. All cell cultures were maintained at 37°C in a 5% CO_2_ atmosphere and 95% humidity.

### Hydrogel preparation

For culturing neurons in 3D, three different hydrogels were used as scaffolds: collagen, HPC and GGC. The gel samples were prepared in a 100 µl volume in 24-well plates with a 13 mm diameter glass bottom (MatTek).

Collagen type I (Col1, rat tail, 3 mg/ml, Gibco, Life Technologies) solution was used to prepare 0.5 mg/ml collagen gels according to the manufacturer's instructions. To prevent premature gelling, all reagents were kept at 4°C during solution preparation, and only four samples were prepared at a time. First, 1 M NaOH, sterile distilled H_2_O and 10×PBS were mixed in a sterile tube. Next, a cell pellet was mixed with 3 mg/ml collagen and the rest of the reagents. After careful mixing, 100 µl of the collagen-cell solution was dispersed to the wells. After gel formation (1 h at 37°C), 1 ml NMM was carefully added on top of the gels.

HPC hydrogel was prepared as previously described for HA1-PVA-Col hydrogel (Ylä-Outinen et al., 2019a). Briefly, gels were prepared with a hyaluronan (HA, Merck KGaA) concentration of 8 mg/ml, polyvinyl alcohol (PVA, Merck KGaA) concentration of 4 mg/ml and collagen concentration of 0.5 mg/ml. Prior to gel preparation, 20 mg/ml HA and 10 mg/ml PVA were prepared as previously described (Karvinen et al., 2018), and the solid components were dissolved in 10% sucrose. The solutions were sterile-filtered and stored at 4°C. The collagen portion for the gels was prepared as described earlier. When preparing the samples, the HA portion was first measured in the wells with a positive displacement pipette. Second, the PVA portion was mixed with the collagen solution and mixed carefully with the cells. Next, the PVA-collagen-cell solution was mixed with the HA portion in the wells. Gelling occurred within seconds. Finally, the gels were incubated for a short period of time at +37°C for further gelling before medium was added.

Spermidine (SPD, Merck KGaA)-crosslinked gellan gum gels (GG) (Gelzan, CP Kelco/Merck KGaA) (Koivisto et al., 2017) were used in combination with collagen in the GGC gels. The final gels consisted of 4.5 mg/ml GG with 3% SPD and 0.3 mg/ml collagen. Prior to gel preparation, the GG was sterile-filtered at 60°C. During solution preparation, GG and SPD were kept at 37°C. Both reagents were prepared in 10% sucrose to reduce the osmotic pressure on the cells. Gel preparation was started by preparing the cells and collagen solution as described earlier. SPD was first pipetted in each well, after which the collagen-cell solution was mixed with the SPD. Finally, the GG portion was added to the wells and mixed quickly with the rest of the reagents. Gelling occurred within seconds. The gels were incubated at 37°C for further gelling, after which medium was added on top of the gels.

### Neuronal exposure to IM

Ionomycin calcium salt (1 mM, Merck KGaA) solution in N3 medium was used for the induction of necrosis in 2-week-old neurons cultured in collagen gel. Five hundred microlitres of the 12.5 µM IM solution was used per sample and cells were incubated in this solution for 6 h at 37°C.

### Inducing neuronal damage with OGD and IM

Neurons were exposed to OGD according to a previously published protocol (Juntunen et al., 2020) after 2 weeks of culturing in collagen gel. The OGD samples were first washed with DMEM without glucose (Thermo Fisher Scientific) for 10 min. Next, the samples were provided with fresh DMEM without glucose and incubated in hypoxic conditions at 37°C with 1% O_2_ for 24 h. The control samples were prepared by washing them with N3 medium for 10 min, changing the medium to NMM, and incubating the samples in NMM for 24 h at 37°C with 21% O_2_. In addition, samples with 12.5 µM IM were prepared as previously described. IM exposure was performed during the last 6 h of the 24 h OGD incubation. After incubation, NMM was replaced with fresh medium for all the samples, which were subsequently re-perfused for 24 h at 37°C with 21% O_2_.

### ICC staining

The previously described ICC staining protocol for neurons in hydrogels (Koivisto et al., 2017) was optimized by prolonging the antibody washing times. The samples were fixed with 4% paraformaldehyde for 60 min. Blocking of nonspecific binding sites was performed with 10% normal donkey serum, 0.1% Triton X-100 and 1% bovine serum albumin (BSA) (all from Merck KGaA) in PBS at room temperature for 60 min. After blocking, the samples were washed once with a solution consisting of 1% normal donkey serum, 0.1% Triton X-100 and 1% BSA in PBS. The same solution was used for primary antibody incubation. Anti-βIII-tubulin chicken IgY (1:100, ab41489, Abcam), anti-βIII-tubulin mouse IgG2b (1:1000, T8660, Merck KGaA), anti-MAP2 chicken IgG (1:2000, NB300-213, Novus Biologicals), anti-MAP2 rabbit (1:600, AB5622, Millipore) and anti-NeuN mouse IgG1 (1:500, MAB377, Millipore) were used for staining. The samples were incubated on a shaker for 2 days at 4°C with 500 µl of the primary antibody mixture. The samples were then washed with 1% BSA in PBS on a shaker for 2 days at 4°C with five repeated washes. Washing solution was changed at 0 h, 1 h, 8 h, 24 h and 32 h. The secondary antibody solution was prepared in 1% BSA in PBS. The secondary antibodies used were Alexa Fluor 488 goat anti-chicken IgG (1:400, A11039), Alexa Fluor 488 donkey anti-mouse IgG (1:400, A21202), Alexa Fluor 568 donkey anti-rabbit IgG (1:400, A10042) and Alexa Fluor 568 donkey anti-mouse IgG (1:400, A10037) (all from Life Technologies). Samples were incubated on a shaker for 24 h at 4°C with 500 µl of the secondary antibody mixture. Next, the samples were incubated with PBS with 4′,6-diamidino-2-phenylindole (DAPI; 1:4000, Merck KGaA) for 3 h at room temperature and washed with PBS. Finally, the samples were mounted with VECTASHIELD (refractive index=1.45, Vector Laboratories) by adding 100 µl of the mounting medium on top of each sample.

### Imaging and image preparation

A Zeiss LSM 780 LSCM confocal microscope (Carl Zeiss AG) with a 25× glycerine immersion objective (NA 0.8) was used to image the samples. A resolution of 0.33 µm×0.33 µm×0.5 µm was used for all the images. After imaging, the 3D stacks were deconvolved with Huygens Essentials software (Scientific Volume Imaging, Hilversum, Netherlands). The deconvolved colour channels were merged using ImageJ (U.S. National Institutes of Health). An Olympus IX51 inverted fluorescence microscope and an Olympus DP30BW digital camera (Olympus Corporation) with a 10× objective (NA 0.30) without immersion medium were used to image the samples in 2D format. ImageJ was used to visualize the 2D images.

### Neurite tracing and analysis

Neuronal tracing and analysis were performed with Imaris 9.3.1 and 9.9.1 (Bitplane) and Avizo 2019.1 (Thermo Fisher Scientific/ZIB). A computer with Intel Core i7-6900 K CPU (3.2 GHz) and an NVIDIA TITAN Xp GPU were used for the analysis with Imaris, and a computer with an Intel Xeon E5-2687W CPU (3.4 GHz) and an NVIDIA Quadro K6000 GPU were used for the analysis with Avizo. Before neuronal tracing, the image stacks were cropped into sizes of 255 µm×255 µm×100 µm.

With Imaris, the Autopath algorithm with an automatic mode for neuronal tracing was used. Before tracing, the neurite channel was merged with the NeuN channel using the ‘Channel Arithmetics’ function. The merged channel was smoothed with a Gaussian filter (filter size: 0.332). For tracing, a 1 µm seed-point diameter (neurite diameter), 12 µm starting-point diameter and 4 µm maximum gap width were used. To quantify the number of neurites originating from the somas (neurites per soma), Sholl analysis was used. A soma region was defined as a sphere with an 8 µm radius, and the number of neurites intersecting with the sphere was quantified. The number of nuclei in the images was quantified using the ‘Spots’ function.

With Avizo, a curvature-driven diffusion filter was used to smooth the neurite surfaces before tracing. An interactive thresholding module was used to binarize the image. The ‘Fill holes’ module was then used to fill cavities caused by hollow regions. The ‘Auto skeleton’ tool was used to trace the neurites from the images. For loop and graph identification, the ‘Filament Editor’ module was used.

### Statistical analysis

Statistical analysis was performed with GraphPad Prism 9.0.0 (GraphPad Software) and IBM SPSS Statistics 28.0.1.0. Kruskal–Wallis and Mann–Whitney *U*-tests were used for the comparison of three different hydrogels in terms of support for neurite growth and gel stability (*n*=10-18/group, from three independent experiments). Spearman's rank correlation test was used for pairwise comparison of the tracing results (*n*=26 separate images from one experiment) acquired with Imaris and Avizo. Unpaired, two-tailed Student's *t*-test was used to define the differences in network lengths of IM-treated and control samples (*n*=8/group, from one experiment). Kruskal–Wallis with Dunn's multiple comparisons test was used to define the statistical significance of differences in network properties among IM-treated, OGD-treated and control samples (*n*=10/group, from two experiments, each sample including two or three technical replicates).

## Supplementary Material

Supplementary information
