# Supplementary material for: Practical guide for preparation, computational reconstruction and analysis of 3D human neuronal networks in control and ischaemic conditions
Source: Development. 2022 Aug 5;149(20):dev200012. doi: 10.1242/dev.200012 (PMC9440753; doi:10.1242/dev.200012)
Supplement: Supplementary information [file develop-149-200012-s1.pdf]

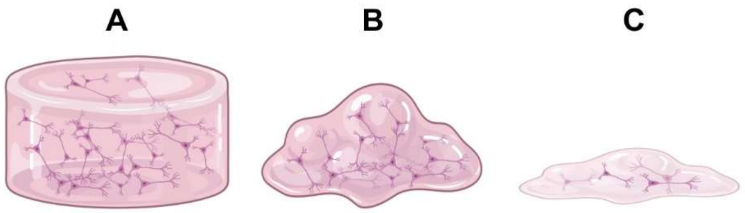

Fig. S1. Examples of hydrogel indexing.

Representative drawings of intact (index = 3) (A), partially degraded (index = 2) (B) and completely degraded (index = 1) (C) hydrogels, observed by eye during staining procedure. Created with BioRender.com.

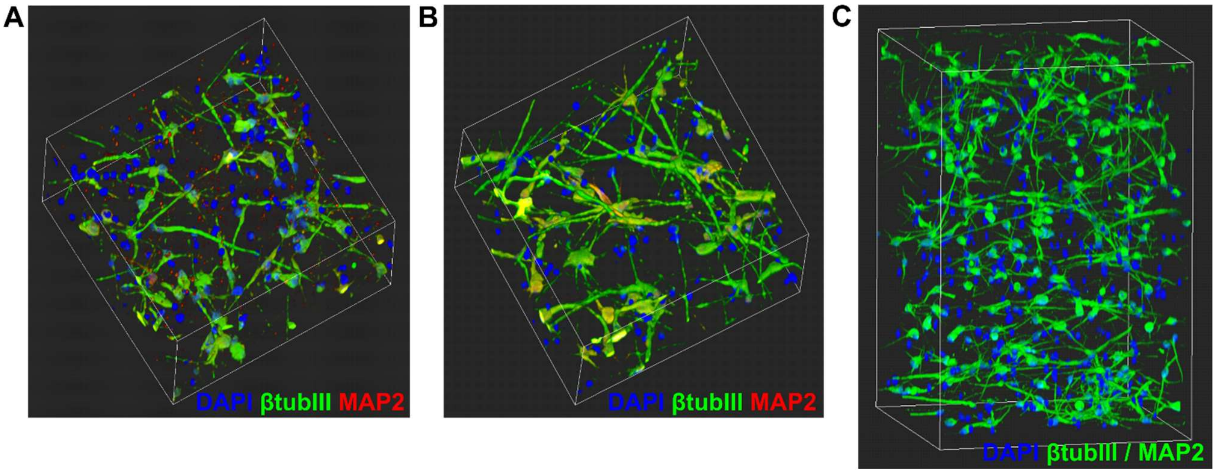

Fig. S2. ICC staining protocol optimization.

Representative image of the original (A) and optimized (B) staining protocols. Bounding box = 255 x 255 x 200 μm. (C) A 3D sample in its full thickness. Bounding box = 340 μm x 340 μm x 500 μm. Images were taken with a Zeiss LSM 780 LSCM confocal microscope system.

Development • Supplementary information

A

B

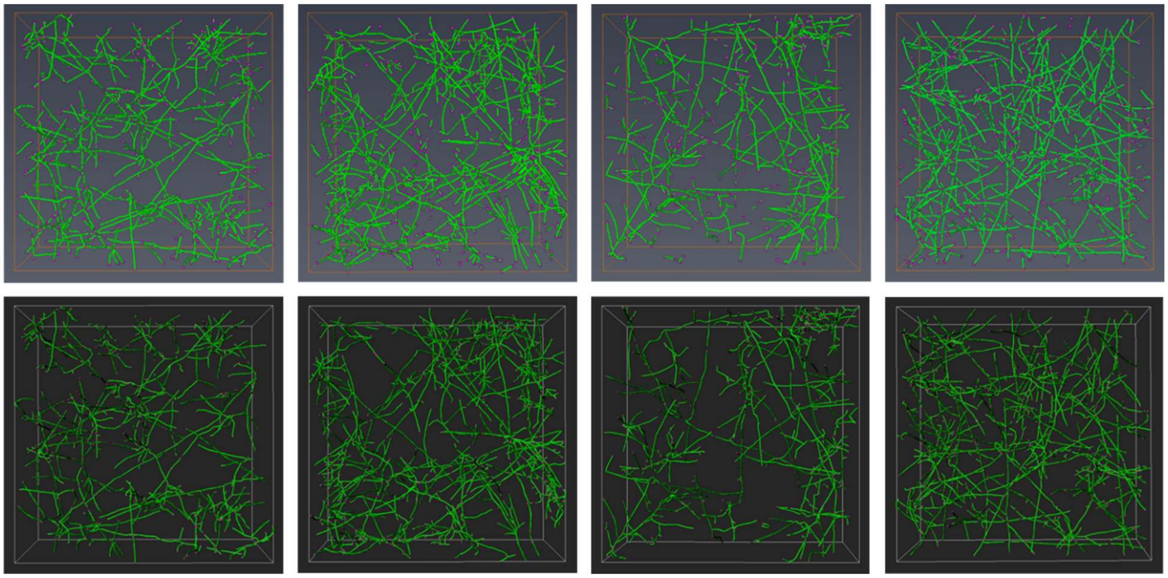

Fig. S3. Comparison of tracing results with Imaris and Avizo. Corresponding tracing results from Avizo (A) and Imaris (B). The traced neurites are labelled in green, and the branching and ending points are labelled in magenta.
